# Prevalence of lung tumors in patients with esophageal squamous cell carcinoma and vice versa: a systematic review and meta-analysis

**DOI:** 10.1007/s00432-022-04103-0

**Published:** 2022-06-23

**Authors:** Laurelle van Tilburg, Steffi E. M. van de Ven, Manon C. W. Spaander, Laurens A. van Kleef, Robin Cornelissen, Marco J. Bruno, Arjun D. Koch

**Affiliations:** 1 Department of Gastroenterology and Hepatology, Erasmus MC Cancer Institute, University Medical Center Rotterdam, Rotterdam, The Netherlands; 2 Department of Pulmonary Medicine, Erasmus MC Cancer Institute, University Medical Center Rotterdam, Rotterdam, The Netherlands

**Keywords:** Second primary tumors, Esophageal cancer, Lung cancer, Squamous cell carcinoma, Oncology, Meta-analysis

## Abstract

**Purpose:**

Recent reports suggest an increased prevalence of lung second primary tumors (LSPTs) in esophageal squamous cell carcinoma (ESCC) patients and vice versa. However, the exact prevalence of SPTs remains unclear and screening for these SPTs is currently not routinely performed in western countries. We aimed to report on the prevalence of LSPTs in patients with ESCC and esophageal second primary tumors (ESPTs) in patients with lung cancer (LC).

**Methods:**

Databases were searched until 25 March 2021 for studies reporting the prevalence of LSPTs in ESCC or vice versa. Pooled prevalences with 95% confidence intervals (CI) of SPTs were calculated with inverse variance, random-effects models and Clopper–Pearson.

**Results:**

Nineteen studies in ESCC patients and 20 studies in LC patients were included. The pooled prevalence of LSPTs in patients with ESCC was 1.8% (95% CI 1.4–2.3%). For ESPTs in LC patients, the pooled prevalence was 0.2% (95% CI 0.1–0.4%). The prevalence of LSPTs in ESCC patients was significantly higher in patients treated curatively compared to studies also including palliative patients (median 2.5% versus 1.3%). This difference was consistent for the ESPT prevalence in LC patients (treated curatively median 1.3% versus 0.1% for all treatments). Over 50% of the detected SPTs were squamous cell carcinomas and were diagnosed metachronously.

**Conclusion:**

Patients with ESCC and LC have an increased risk of developing SPTs in the lungs and esophagus. However, the relatively low SPT prevalence rates do not justify screening in these patients. Further research should focus on risk stratification to identify subgroups of patients at highest risk of SPT development.

**Supplementary Information:**

The online version contains supplementary material available at 10.1007/s00432-022-04103-0.

## Introduction

Over half a million esophageal cancers and 2 million lung cancers (LC) were diagnosed worldwide in 2018 (Arnold et al. 2020; Bray et al. 2018; Lu et al. 2019). The major risk factor for esophageal squamous cell carcinoma (ESCC) and LC is tobacco smoking (Freedman et al. 2016). The prognosis of both cancers remains poor, although the 5-year survival rate has improved to approximately 22% for ESCC in 2018 and 23% for LC in 2020 (Putten et al. 2018; State of Lung Cancer 2021). The poor survival rates of patients with ESCC and LC could partially be explained by the occurrence of second primary tumors (SPTs) (Lu et al. 2019; Ven et al. 2019, 2020).

For patients with ESCC, the occurrence of SPTs is frequently explained by the theory of field cancerization (Slaughter et al. 1953). This theory states that chronic exposure of the epithelium surrounding the primary tumor to carcinogens, especially tobacco, can lead to (pre)malignant changes of the epithelium. Most SPTs in patients with ESCC are located in the upper aero-digestive tract, especially in the head and neck region and lungs (Ven et al. 2020).

Large incidence differences for both ESCC and LC exist worldwide, with high incidence rates of both cancers reported in Eastern Asia (Bray et al. 2018). However, little is known regarding the prevalence of LSPTs and ESPTs in this patient population, especially in non-Asian countries. Moreover, the potential yield and benefit of screening for SPTs in patients with ESCC and LC remains unclear.

Nowadays, screening for LSPTs in patients with ESCC and esophageal second primary tumors (ESPTs) in patients with LC is not routinely implemented in Western countries (guideline non-small cell lung cancer 2021; Guideline esophageal cancer 2021; Guideline small cell lung carcinoma 2021). According to current Asian guidelines, a trachea-bronchoscopy to detect SPTs is advised during the diagnostic workup in all patients with ESCC with chronic alcohol and tobacco consumption (Lordick et al. 2016; Muro et al. 2019). The Dutch guidelines suggest screening for LSPTs in ESCC patients may be considered and does not mention screening for ESPTs in patients with LC (Guideline esophageal cancer 2021).

The primary objective of this systematic review and meta-analysis is to investigate the prevalence of LSPTs in patients with ESCC and the prevalence of ESPTs in patients with LC. The secondary objectives are to assess the tumor stage of SPTs and time interval between the primary cancer diagnosis and detection of SPTs.

## Materials and methods

### Search strategy

The databases PubMed, Embase, Medline, Cochrane Central, Google Scholar, and Web of Science were searched by two independent investigators (L.T. and S.V.) until 25 March 2021. The systematic search contained keywords for second/multiple primary tumor, esophageal cancer and lung cancer. No time restrictions were set. The search was performed in collaboration with the medical library of the Erasmus University Rotterdam, the Netherlands. The complete search strategy is available in Supplementary Appendix 1. In addition, reference lists of included studies were searched to identify additional relevant studies.

### Study inclusion

Studies that reported the proportion of LSPTs (of all histological types) in patients with ESCC or the proportion of ESPTs (both ESCC and esophageal adenocarcinoma) in patients with LC were included. Studies without original data, case reports, non-human and non-English studies were excluded. Two independent investigators (L.T. and S.V.) screened titles and abstracts followed by full texts of potentially eligible articles identified by the search strategy. In case of any disagreement, a consensus was reached through discussion (with L.T., S.V., and A.K.). The Preferred Reporting Items for Systematic Reviews and Meta-Analyses (PRISMA) flowchart was used to create an overview of the data screening process (Moher et al. 2009).

### Data extraction and quality assessment

The extracted information from each study included: study characteristics (author, year of publication, study country, design, and setting) and patient characteristics (gender, number of patients with ESCC and LSPTs, number of patients with LC and ESPTs, time interval between the primary cancer diagnosis and detection of SPTs, tumor stage, histopathology, and treatment). The methodological quality of each study was assessed with the Newcastle–Ottawa scale for quality assessment for cohort studies (Wells et al. 2000). Funnel plots and Egger tests were used to assess the risk of publication bias (Duval and Tweedie 2000).

### Outcomes and definitions

The primary outcomes were (1) the pooled prevalence of LSPTs in patients with ESCC and (2) the pooled prevalence of ESPTs in patients with LC. Secondary outcomes included the tumor stage of SPTs and the time from the diagnosis of the primary cancer to the detection of an SPT. The criteria for SPTs from Warren and Gates were used; an SPT must be (1) a malignant tumor based on histopathological assessment, (2) separated from the primary cancer by normal mucosa, and (3) the possibility of the SPT being a recurrence or metastasis from the primary cancer must be ruled out (Warren 1932). The time to the detection of SPTs was classified as a tumor in the history before the diagnosis of ESCC or LC and synchronous and metachronous SPTs (Cahan et al. 1976). Synchronous SPTs were defined as the detection of an SPT within 6 months of the diagnosis of the primary tumor (this may be referred to as simultaneous). Metachronous SPTs were defined as the detection of an SPT at least 6 months after the diagnosis of the primary tumor.

### Data analysis

For the meta-analysis, the SPT prevalence was calculated for each study as the number of SPTs divided by the number of the patient population in that specific study. The heterogeneity between included studies was assessed using the inconsistency index (*I*^2^). The incidence of both ESCC and LC differs strongly worldwide, with the highest incidence rates of both cancers reported in Eastern Asia (Bray et al. 2018). Therefore, the random-effects model with inverse variance was used to calculate the pooled prevalence and 95% confidence intervals (CI) were calculated with Clopper–Pearson. Excessive influence of individual studies on the pooled prevalence was investigated in sensitivity analyses. Standardized incidence ratios (SIRs) of the included studies were extracted for a comparison with the risk in the general population to develop lung cancer or esophageal cancer. Data were presented as counts with percentages. Analyses were performed in R version 4.1.1 (The R Foundation Statistical Computing, Vienna, Austria) with meta version 4.18-2 and metafor version 3.0-2. All tests were performed two-sided and *P* < 0.05 was considered significant.

## Results

### Study selection and quality assessment

**Fig. 1:**
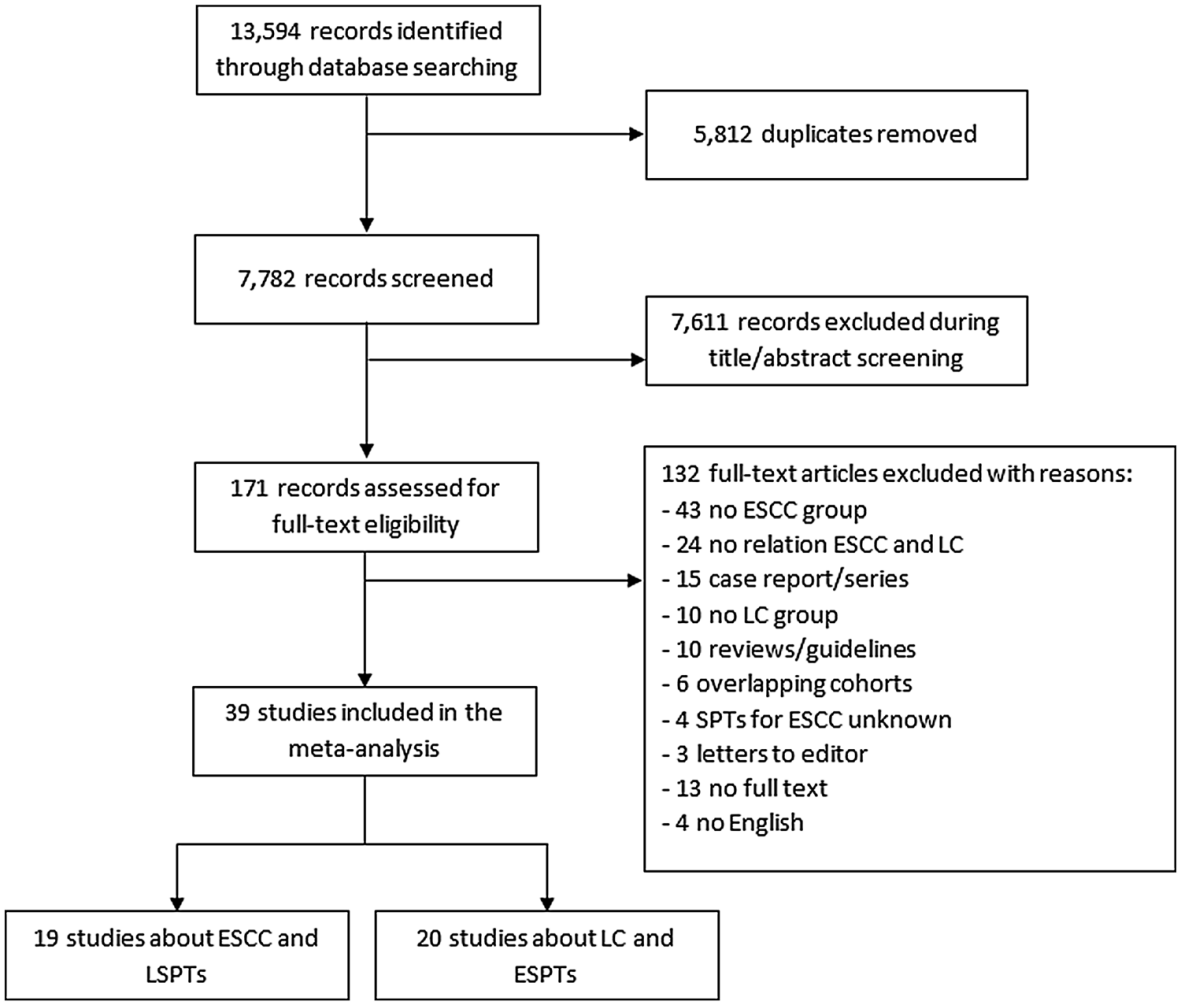
Flowchart of study inclusion. *ESCC* esophageal squamous cell carcinoma; *ESPT* esophageal second primary tumor; *LC* lung cancer; *LSPT* lung second primary tumor

The literature search identified 13,594 records (shown in Fig. 1). After removing duplicates, 7,782 articles were assessed for titles and abstracts, of which 171 articles were potentially eligible. After full-text reviewing, 39 studies were included in this systematic review and meta-analysis. The quality assessment according to the Newcastle–Ottawa Scale of included studies is shown in Supplementary Table 1.

### Study characteristics

The 39 included studies consisted of 19 studies performed in patients with ESCC (Supplementary Table 2) (Ven et al. 2020; Poon et al. 1998; Motoyama et al. 2003; Yoshida et al. 2020; Hu et al. 2015; Lee et al. 2013; Yamaguchi et al. 2018; Otowa et al. 2016; Natsugoe et al. 2005; Kumagai et al. 2001; Kokawa et al. 2001; Nagasawa et al. 2000; Voormolen et al. 1995; Fekete et al. 1994; Chen et al. 2019; Chuang et al. 2008; Ribeiro Júnior et al. 1999; Fogel et al. 1985; Fitzpatrick et al. 1984) and 20 studies performed in patients with LC (Supplementary Table 3) (Abdel-Rahman and Cheung 2017; Chuang et al. 2010; Coyte et al. 2014; Duchateau and Stokkel 2005; Faehling et al. 2018; Haraguchi et al. 2007; Hsieh et al. 1997; Kaneko and Yamaguchi 1999; Kawahara et al. 1998; Komatsu et al. 2019; Levi et al. 1999; Li et al. 2015; Reinmuth et al. 2013; Shan et al. 2017; Son et al. 2013; Su et al. 2017; Takigawa et al. 2006; Teppo et al. 2001; Shimizu et al. 2001; Fink-Neuboeck et al. 2020). The studies comprised a total of 62,924 patients with ESCC (median 601, range 185–30,121) and 648,315 patients with LC (median 4111, range 32–258,559). Twenty-two studies were performed in Asian countries (Poon et al. 1998; Motoyama et al. 2003; Yoshida et al. 2020; Hu et al. 2015; Lee et al. 2013; Yamaguchi et al. 2018; Otowa et al. 2016; Natsugoe et al. 2005; Kumagai et al. 2001; Kokawa et al. 2001; Nagasawa et al. 2000; Haraguchi et al. 2007; Hsieh et al. 1997; Kaneko and Yamaguchi 1999; Kawahara et al. 1998; Komatsu et al. 2019; Li et al. 2015; Shan et al. 2017; Son et al. 2013; Su et al. 2017; Takigawa et al. 2006; Shimizu et al. 2001), ten studies in Europe (Ven et al. 2020; Voormolen et al. 1995; Fekete et al. 1994; Coyte et al. 2014; Duchateau and Stokkel 2005; Faehling et al. 2018; Levi et al. 1999; Reinmuth et al. 2013; Teppo et al. 2001; Fink-Neuboeck et al. 2020) and 7 studies in other countries (Chen et al. 2019; Chuang et al. 2008, 2010; Ribeiro Júnior et al. 1999; Fogel et al. 1985; Fitzpatrick et al. 1984; Abdel-Rahman and Cheung 2017). Most studies were performed retrospectively (Ven et al. 2020; Yoshida et al. 2020; Hu et al. 2015; Lee et al. 2013; Yamaguchi et al. 2018; Otowa et al. 2016; Natsugoe et al. 2005; Kumagai et al. 2001; Kokawa et al. 2001; Nagasawa et al. 2000; Voormolen et al. 1995; Fekete et al. 1994; Chen et al. 2019; Chuang et al. 2008, 2010; Ribeiro Júnior et al. 1999; Fogel et al. 1985; Fitzpatrick et al. 1984; Abdel-Rahman and Cheung 2017; Coyte et al. 2014; Duchateau and Stokkel 2005; Faehling et al. 2018; Haraguchi et al. 2007; Hsieh et al. 1997; Kaneko and Yamaguchi 1999; Kawahara et al. 1998; Komatsu et al. 2019; Levi et al. 1999; Li et al. 2015; Reinmuth et al. 2013; Shan et al. 2017; Son et al. 2013; Su et al. 2017; Takigawa et al. 2006; Teppo et al. 2001). Four studies were performed prospectively (Poon et al. 1998; Motoyama et al. 2003; Shimizu et al. 2001; Fink-Neuboeck et al. 2020), of which two were screening studies to detect SPTs (Motoyama et al. 2003; Shimizu et al. 2001). The funnel plots and Egger tests showed no proof of publication bias for the prevalence of LSPTs in patients with ESCC (*P* = 0.11) and the prevalence of ESPTs in patients with LC (*P* = 0.16) (Supplementary Fig. 1).

### Prevalence of LSPTs

**Fig. 2:**
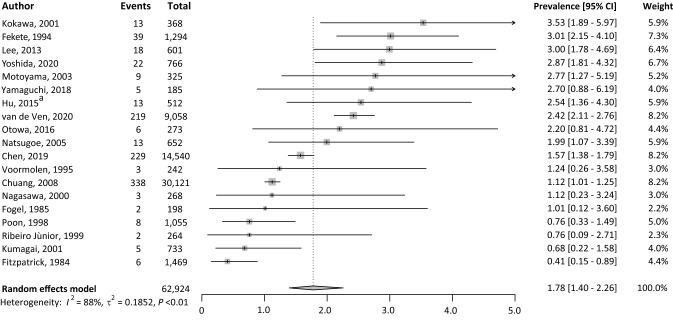
Overview of the prevalence of LSPTs in patients with ESCC. *CI* confidence interval; *ESCC*, esophageal squamous cell carcinoma; *LSPT* lung second primary tumor; *I*^2^ inconsistency index; *τ*^2^ tau-squared represents the extent of variation among the effects observed in different studies. ^a^Hu et al. excluded all lung squamous cell carcinoma (*n* = 11), which occurred within the first 5 years after the diagnosis of ESCC, as potential LSPTs

**Fig. 3:**
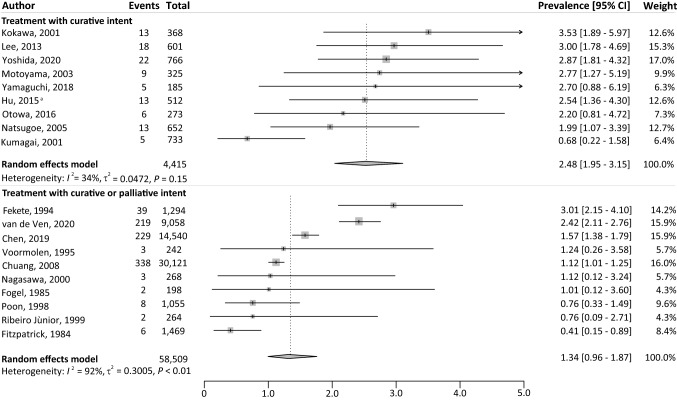
Overview of the prevalence of LSPTs in patients with ESCC for different treatment intents. *CI* confidence interval; *ESCC* esophageal squamous cell carcinoma; *LSPT* lung second primary tumor; *I*^2^, inconsistency index; *τ*^2^ tau-squared represents the extent of variation among the effects observed in different studies. ^a^Hu et al. excluded all lung squamous cell carcinoma (*n* = 11), which occurred within the first 5 years after the diagnosis of ESCC, as potential LSPTs

The pooled prevalence of LSPTs in patients with ESCC was 1.8% (95% CI 1.4–2.3%) with a high level of heterogeneity (*I*^2^ = 88%, *P* < 0.01) (Fig. 2). In total, 953 LSPTs were detected in 62,924 patients with ESCC. The pooled prevalence of LSPTs was significantly higher among ESCC patients treated with curative intent (2.5%, 95% CI 2.0–3.2%), compared to studies that also included palliative ESCC patients (1.3%, 95% CI 1.0–1.9%) (Fig. 3). Sub analyses with only patients treated with palliative care were not possible because LSPT rates specifically for palliative ESCC patients were not reported in the included studies. The LSPT prevalence was suggestively higher in ESCC patients from Asian countries (2.1%, 95% CI 1.6–2.8%) compared to non-Asian countries (1.5%, 95% CI: 1.0–2.1%) (Supplementary Fig. 2) and for studies published in the last decade (2010–2021 2.3%, 95% CI 1.8–3.0%) compared to previous decades (before 2000 1.0%, 95% CI 0.4–2.3%, 2000–2010 1.7%, 95% CI 1.0–2.8%) (Supplementary Fig. 3). However, no statistically significant differences could be demonstrated.

### Characteristics and time to diagnosis of LSPTs

Most patients with ESCC that developed LSPTs were male (98.3%) (Lee et al. 2013; Fekete et al. 1994; Ribeiro Júnior et al. 1999). The tumor stage of LSPTs was stage 0–I (*n* = 20, 43.5%), stage II–III (*n* = 9, 19.6%), and stage IV (*n* = 17, 37.0%) in three retrospective studies (Yamaguchi et al. 2018; Fekete et al. 1994; Ribeiro Júnior et al. 1999). In one screening study, 6/8 LSPTs were detected in asymptomatic patients of which five LSPTs were detected in early and curable stages (Motoyama et al. 2003). Based on four studies, the histology of the LSPTs was squamous cell carcinoma in 38–100% of the LSPTs per study (total 51/69), adenocarcinoma in 10–56% (total 13/69), small cell carcinoma in 0–6% (total 3/69) and adenosquamous carcinoma in 0–11% (1/69) (Motoyama et al. 2003; Lee et al. 2013; Fekete et al. 1994; Fogel et al. 1985). The time to detection of LSPTs was reported in 16 studies (Table 1) (Ven et al. 2020; Poon et al. 1998; Yoshida et al. 2020; Lee et al. 2013; Otowa et al. 2016; Natsugoe et al. 2005; Kumagai et al. 2001; Kokawa et al. 2001; Voormolen et al. 1995; Fekete et al. 1994; Chuang et al. 2008; Ribeiro Júnior et al. 1999; Fogel et al. 1985; Fitzpatrick et al. 1984). The study of Fitzpatrick et al. combined lung tumors before ESCC diagnosis with synchronous LSPTs (Fitzpatrick et al. 1984). Natsugoe et al. reported lung tumors before ESCC diagnosis and metachronous LSPTs together (Natsugoe et al. 2005). The studies of Yamaguchi et al. and Motoyama et al. only reported metachronous LSTPs (Motoyama et al. 2003; Yamaguchi et al. 2018). Among 12 studies, comprising 44,973 patients with ESCC, LSPTs were detected synchronously in 198/675 patients and metachronously in 225/675 patients. In 11 studies, 252/456 patients with ESCC had an history of lung cancer (Poon et al. 1998; Lee et al. 2013; Kumagai et al. 2001; Kokawa et al. 2001; Voormolen et al. 1995; Fekete et al. 1994; Ribeiro Júnior et al. 1999; Fogel et al. 1985; Fitzpatrick et al. 1984).

**Table 1:** Follow-up time for the detection of lung tumors in patients with ESCC

References	Total LSPTs, *n*	History of LC, *n* (%)	Synchronous LSPTs, *n* (%)	Metachronous LSPTs, *n* (%)	Time from LC in history to ESCC	Time to detection of metachronous LSPTs
Ven et al. (2020)	219	–	123 (56.2)	96 (43.8)	–	Median 3.2 year (IQR 1.9–4.5)
Yoshida et al. (2020)	22	4 (18.2)	2 (9.1)	16 (72.7)	NR	NR
Yamaguchi et al. (2018)	5	–	–	5 (100.0)	–	NR
Otowa et al. (2016)	6	4 (66.7)	2 (33.3)	0	NR	–
Lee et al. (2013)	18	1 (5.6)	9 (50.0)	8 (44.4)	NR	NR
Chuang et al. (2008)	338	226 (66.9)	30 (26.8)	82 (73.2)	< 12 months *n* = 62 1–4 year *n* = 83 5–9 year *n* = 48 ≥ 10 year *n* = 33	6–11 months *n* = 6 1–4 year *n* = 43 ≥ 5 year *n* = 33
Motoyama et al. (2003)	9	–	–	9 (100.0)	–	Reported for 5 patients: 12, 14, 20, 23, 43 and 112 months
Kokawa et al. (2001)	13	2 (15.4)	4 (30.8)	7 (53.8)	NR	Mean 23 months (sd 10.4)
Kumagai et al. (2001)	5	1 (20.0)	3 (60.0)	1 (20.0)	NR	NR
Ribeiro Júnior et al. (1999)	2	1 (50.0)	0	1 (50.0)	2 years	6 year
Poon et al. (1998)	8	4 (50.0)	2 (25.0)	2 (25.0)	NR	NR
Voormolen et al. (1995)	3	1 (33.3)	1 (33.3)	1 (33.3)	NR	NR
Fekete et al. (1994)	39	7 (17.9)	22 (56.4)	10 (25.6)	Mean 46 months (range 18–77)^a^	
Fogel et al. (1985)	2	1 (50.0)	0	1 (50.0)	84 months	21 months
Total	**675**	**252**	**198**	**239**		

*ESCC* esophageal squamous cell carcinoma, *IQR* interquartile range, *LC* lung cancer, *LSPT* lung second primary tumor, *NR* not reported, *sd* standard deviation

^a^Time interval between the diagnosis of ESCC and the diagnosis of LC

### Characteristics of ESCC

Twelve studies reported the tumor stage of ESCC (Ven et al. 2020; Poon et al. 1998; Yoshida et al. 2020; Hu et al. 2015; Lee et al. 2013; Yamaguchi et al. 2018; Otowa et al. 2016; Natsugoe et al. 2005; Kumagai et al. 2001; Kokawa et al. 2001; Nagasawa et al. 2000; Chen et al. 2019). However, only the study of Lee et al. reported the numbers of LSPTs for each ESCC tumor stage (Lee et al. 2013). In this study, 6 LSPTs were detected in 172 patients with ESCC stage 0–I, 3 LSPTs in 136 patients with ESCC stage II, 4 LSPTs in 118 patients with ESCC stage III and 1 LSPT in five patients with ESCC stage IV (Lee et al. 2013). In the included studies, treatments for patients with ESCC were surgery (*n* = 13,915), chemo-or-radiotherapy (*n* = 15,071) and endoscopic resection (*n* = 275) (Ven et al. 2020; Poon et al. 1998; Motoyama et al. 2003; Yoshida et al. 2020; Hu et al. 2015; Lee et al. 2013; Yamaguchi et al. 2018; Otowa et al. 2016; Natsugoe et al. 2005; Kumagai et al. 2001; Kokawa et al. 2001; Nagasawa et al. 2000; Voormolen et al. 1995; Chen et al. 2019; Chuang et al. 2008; Ribeiro Júnior et al. 1999; Fogel et al. 1985). Nine studies only included patients with ESCC treated with curative intent (Motoyama et al. 2003; Yoshida et al. 2020; Hu et al. 2015; Lee et al. 2013; Yamaguchi et al. 2018; Otowa et al. 2016; Natsugoe et al. 2005; Kumagai et al. 2001; Kokawa et al. 2001). The follow-up time of patients with ESCC was not reported in eight studies and median shorter than 1.5 years after ESCC diagnosis in two studies (Yoshida et al. 2020; Kumagai et al. 2001; Kokawa et al. 2001; Nagasawa et al. 2000; Fekete et al. 1994; Chen et al. 2019; Ribeiro Júnior et al. 1999; Fogel et al. 1985).

### Prevalence of ESPTs

**Fig. 4:**
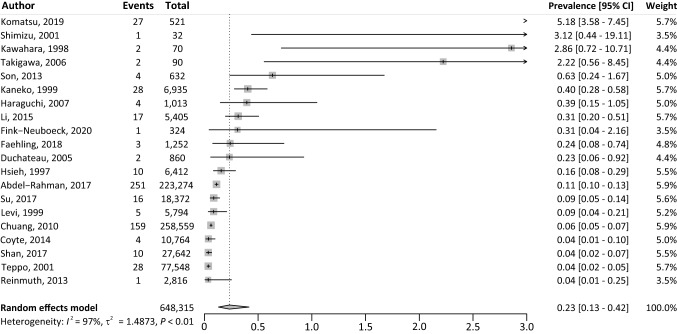
Overview of the prevalence of ESPTs in patients with LC. *CI* confidence interval; *ESPT* esophageal second primary tumor; *LC* lung cancer; *I*^2^ inconsistency index; *τ*^2^ tau-squared represents the extent of variation among the effects observed in different studies

**Fig. 5:**
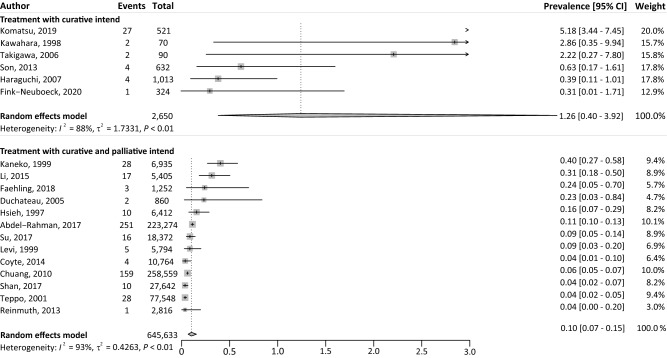
Overview of the prevalence of ESPTs in patients with LC for different treatment intents. *CI* confidence interval; *ESPT* esophageal second primary tumor; *LC* lung cancer; *I*^2^ inconsistency index; *τ*^2^, tau-squared represents the extent of variation among the effects observed in different studies

The pooled prevalence of ESPTs in patients with LC was 0.2% (95% CI 0.1–0.4%) with significant heterogeneity (*I*^2^ = 97%, *P* < 0.01) (Fig. 4). In total, 575 ESPTs occurred in 648,315 patients. The prevalence of ESPTs was significantly higher among patients with LC treated with curative intent (1.3%, 95% CI 0.4–3.9%), compared to studies that also included patients with LC treated with palliative intent (0.1%, 95% CI 0.1–0.2%) (Fig. 5). The ESPT prevalence in LC patients was significantly higher in Asian countries (0.5%, 95% CI 0.2–1.5%), compared to non-Asian countries (0.1%, 95% CI 0.1–0.1%) (Supplementary Fig. 4). No trends were observed in ESPT prevalence for studies published between the last decade, compared to previous decades (Supplementary Fig. 5). Sensitivity analyses did not reveal excessive influence of individual studies on the pooled prevalence (Supplementary Fig. 6).

### Characteristics and time to diagnosis of ESPTs

Based on six studies, 79.3% of the patients with LC that developed ESPTs were male (Abdel-Rahman and Cheung 2017; Chuang et al. 2010; Haraguchi et al. 2007; Kawahara et al. 1998; Su et al. 2017; Teppo et al. 2001; Shimizu et al. 2001). The study of Shimizu et al. (2001) only included male veterans (Shimizu et al. 2001). The tumor stage of ESPTs was known in three studies (Abdel-Rahman and Cheung 2017; Shimizu et al. 2001; Fink-Neuboeck et al. 2020); the ESPTs (*n* = 97) detected in the study of Abdel-Rahman were stage I in 39.2%, stage II in 23.7%, stage III in 12.3%, and stage IV in 24.7% (Abdel-Rahman and Cheung 2017). The screening study of Shimizu performed esophageal screening with Lugol’s chromoendoscopy in 32 patients with LC and detected one early-stage ESPT (Shimizu et al. 2001). In four studies, the histology of ESPTs was squamous cell carcinoma 59–100% of the ESPTs per study (164/267 in total) and adenocarcinoma in 25–31% of ESPTs (78/267 in total) (Abdel-Rahman and Cheung 2017; Hsieh et al. 1997; Kawahara et al. 1998; Son et al. 2013). The time to detect an SPT was noted in 13 studies. Two studies combined history of EC with metachronous ESPTs (Li et al. 2015; Fink-Neuboeck et al. 2020) and another two studies reported on a history of EC and subsequent ESPTs (Coyte et al. 2014; Duchateau and Stokkel 2005). The remaining nine studies reported 87 ESPTs that were detected synchronously and 223 ESPTs metachronously (Table 2) (Abdel-Rahman and Cheung 2017; Coyte et al. 2014; Duchateau and Stokkel 2005; Faehling et al. 2018; Haraguchi et al. 2007; Hsieh et al. 1997; Li et al. 2015; Shan et al. 2017; Son et al. 2013).

**Table 2:** Follow-up time for the detection of esophageal tumors in patients with LC

References	Total ESPTs, *n*	History of EC, *n* (%)	Synchronous ESPTs, *n* (%)	Metachronous ESPTs, *n* (%)
Faehling et al. (2018)	3	3 (100.0)	0	
Abdel-Rahman and Cheung (2017)	251^a^	–	50 (20.1)^b^	199 (79.9)
Shan et al. (2017)	10	10 (100.0)	0	–
Su et al. (2017)	16	–	–	16 (100.0)
Son et al. (2013)	4	1 (25.0)	0	3 (75.0)
Haraguchi et al. (2007)	4	–	3 (75.0)	1 (25.0)
Kaneko and Yamaguchi (1999)	28	–	28 (100.0)	–
Kawahara et al. (1998)	2	–	–	2 (100.0)
Hsieh et al. (1997)	10	2 (20.0)	6 (60.0)	2 (20.0)
Total	**328**	**16**	**87**	**223**

*EC* esophageal carcinoma, *IQR* interquartile range, *LC* lung cancer, *NR* not reported, *sd* standard deviation

^a^For 2 esophageal tumors was the time to detection unknown

^b^Synchronous ESPTs were defined as esophageal cancer occurring within 1 year of LC diagnosis

### Characteristics of LC

The tumor stage of LC was reported in five studies (Abdel-Rahman and Cheung 2017; Faehling et al. 2018; Reinmuth et al. 2013; Takigawa et al. 2006; Fink-Neuboeck et al. 2020); however, none of these studies reported the number of ESPTs for each LC tumor stage. In six studies, only patients with LC treated with curative intent were included. Haraguchi et al. (2007), Komatsu et al. (2019), Son et al. (2013), Takigawa et al. (2006), Shimizu et al. (2001), and Fink-Neuboeck et al. (2020) reported treatments for LC were surgery (*n* = 61,356) and chemo-or-radiotherapy (*n* = 108,961).

### Increased standardized incidence ratios compared to general population

Table 3 shows the studies that reported SIRs for the risk of SPTs, compared to the risk of esophageal or LC in the general population (Ven et al. 2020; Hu et al. 2015; Chen et al. 2019; Chuang et al. 2008, 2010; Abdel-Rahman and Cheung 2017; Levi et al. 1999; Su et al. 2017; Teppo et al. 2001). In all four studies in ESCC patients, a significantly increased risk for LSPTs was reported compared to the general population (Ven et al. 2020; Hu et al. 2015; Chen et al. 2019; Chuang et al. 2008). In five studies performed in patients with LC, SIRs ranging from 1.45 to 2.40 were reported. The study of Abdel-Rahman and Cheung 2017 reported a significantly increased risk for ESPTs in patients with LC, whereas the smaller studies of Su et al. 2017 and did not Levi et al. 1999.

**Table 3:** Standardized incidence ratios (SIRs) for lung second primary tumors (LSPTs) and esophageal second primary tumors (ESPTs)

	Author (year)^ref^	Observed (*n*)	Expected (*n*)	SIR (95% CI) total	SIR (95% CI) males	SIR (95% CI) females	SIR (95% CI) time frames
LSPTs in ESCC	Ven et al. (2020)	123	19	6.42 (5.02–8.06^b^)	5.35 (3.90–7.14^b^)	9.48 (6.29–13.66^b^)	NR
	Chen et al. (2019)	229	63	3.63 (3.17–4.13)	NR	NR	NR
	Hu et al. (2015)^a^	13	5	2.79 (1.60–4.87)	NR	NR	NR
	Chuang et al. (2008)	112	72	1.55 (1.28–1.87)	NR	NR	< 6 mo: 1.47 (0.99–2.10) 6–11 mo: 0.60 (0.22–1.31) 1–4 year: 1.98 (1.43–2.67) ≥ 5 year: 1.64 (1.13–2.31)
ESPTs in LC	Abdel-Rahman and Cheung (2017)	251	105	2.40 (1.62–3.43)	RT: 3.60 (2.77–4.61) No RT: 2.05 (1.65–2.53)	RT: 5.52 (3.50–8.28) No RT: NR	1–5 year 3.09 (1.85–4.82) 5–9 year: 2.13 (0.92–4.19) ≥ 10 year: 1.17 (0.24–3.42)
	Su et al. (2017)	16	11.05	1.45 (0.83–2.35)	1.55 (0.89–2.52)	0.00 (0.00–4.90)	NR
	Teppo et al. (2001)	28	NR	NR	1.23 (0.80–1.79)	0.93 (0.11–3.35)	NR
	Chuang et al. (2010)	159	NR	NR	SCC: 1.78 (1.44–2.18) SCLC: 1.46 (0.75–2.55) Adeno: 1.91 (1.26–3.09)	SCC: 3.31 (1.81–5.56) SCLC: 3.30 (1.21–7.18) Adeno: 1.72 (0.69–3.55)	NR
	Levi et al. (1999)	5	2.8	(0.6–4.4)	NR	NR	NR

*CI* confidence interval, *ESCC* esophageal squamous cell carcinoma, *ESPT* esophageal second primary tumor, *LC* lung cancer, *LSPT* lung second primary tumor, *RT* radiotherapy, *SIR* standardized incidence ratio, *NR* not reported

^a^Hu et al. excluded all lung squamous cell carcinoma (*n* = 11), which occurred within the first 5 years after the diagnosis of ESCC, as potential LSPTs

^b^99% confidence interval

## Discussion

To the best of our knowledge, this is the first systematic review reporting on the prevalence of SPTs in the esophagus and lungs in patients with ESCC and LC. We found a pooled prevalence of LSPTs of 1.8% in patients with ESCC and a prevalence of ESPTs of 0.2% in patients with LC. More than 50% of the detected SPTs were squamous cell carcinomas and were diagnosed metachronously.

The prevalence rates of SPTs in patients with ESCC and LC in this meta-analysis are most likely an underestimation of the actual prevalence of LSPTs in patients with ESCC and vice versa for the following reasons. First, the overall survival rates of patients with ESCC and LC remain poor, although they have increased during the recent decades (Lu et al. 2019; Putten et al. 2018). In 23 of 39 studies, patients treated with palliative intent were also included, while these patients are known to have a median survival of 22 weeks for ESCC and 20 weeks for LC (Lu et al. 2019; Putten et al. 2018). This short life span after the diagnosis of the primary tumor limits the risk for SPT development, while patients treated with curative intent are known to have better survival rates and, therefore, the cumulative risk of SPT development increases over time. This survival bias is also supported by our finding that patients treated with curative intent are significantly more at risk of developing LSPTs and ESPTs than patients who received palliative care. One can hypothesize that the cumulative SPT risks increase in the future, if treatment and survival rates of patients with ESCC and LC may continue to rise.

Second, we found a higher prevalence of LSPTs in patients with ESCC than the prevalence of ESPTs in patients with LC. This difference could be partly explained by the differential use of the positron emission tomography/computed tomography (PET/CT) scan, which is nowadays part of the standard diagnostic workup up of ESCC and LC to detect metastasis (Guideline non small cell lung cancer 2021; Guideline esophageal cancer 2021). Contrary to the high sensitivity of the PET/CT for the detection of early LC, the sensitivity of the PET/CT for the detection of early-stage esophageal cancers is only 38% and is inferior to endoscopic screening for ESPTs (Guideline non small cell lung cancer 2021; Su et al. 2020). Presumably, most ESPTs in patients with LC remained undetected until they reach symptomatic advanced stages, which often cannot be treated with a curative intent. If screening for ESPTs for specific subgroups of patients with LC would ever be considered, an upper gastrointestinal endoscopy would be the examination of choice.

Third, almost all included studies were performed retrospectively, which hampers accurate differentiation between LSPTs and lung metastases of primary ESCC. This difficulty resulted in conservative definitions of LSPTs, e.g., one study choose to exclude all lung squamous cell carcinoma detected within the first 5 years after the diagnosis of ESCC as potential SPTs (Hu et al. 2015) and another only included squamous cell lung carcinoma as LSPTs when the tumors showed clear histologic differences (Motoyama et al. 2003).

In our systematic review, nine included studies reported standardized incidence ratios (SIRs) to develop LSPTs or LSPTs. Most of these studies reported increased SIRs, supporting that SPT prevalence rates found in this study exceed the risk to develop EC and LC in the general population. However, for an adequate comparison with the risk among the general population, matching of all individual patient data of the included studies for parameters, including age, gender, comorbidities, follow-up time and alcohol and tobacco use would be essential.

The SPT prevalence rates found in this meta-analysis currently do not support screening for LSPTs and ESPTs. Future research should focus on identification of subgroups of patients with ESCC and LC with the highest risks for SPT development. Although evidence is limited, patient characteristics with the highest risk for SPTs that can be considered are for example males with chronic tobacco use and early and curable primary tumors. In these patients, the occurrence of SPTs can have major consequences for treatment and prognosis, and screening might potentially be beneficial. Moreover, geographic differences in the incidence of ESCC, LC, and SPTs are an important differentiator in the process of identification of patients with highest risks to develop SPTs. Another issue with regard to screening that needs to be addressed is the optimal timing to screen for SPTs in these patients. This needs to be balanced, between as early as possible to detect SPT at an early and curable stage on one hand and screening of selected patients with improved survival rates on the other hand.

Recently, a large-scale screening study was performed to detect lung cancers among a population of heavy (ex) smokers (Koning et al. 2020). In this study, patients underwent a minimum of 10 years of screening and follow-up with CTs at baseline, year 1, year 3, and year 5.5. The incidence of LC was 5.6%, and screening successfully reduced LC-related mortality. With our findings, combined with the fact that 80–90% of ESCC patients are heavy (ex) smokers (Gruner et al. 2020), one might hypothesize that a subgroup of patients with ESCC would also potentially benefit from CT screening during the ESCC follow-up to detect LSPTs.

Although this systematic review included all available studies reporting on the prevalence of LSPTs and ESPTs, several limitations need to be discussed: (1) different definitions for the diagnosis and timing for SPTs were used. Synchronous and metachronous SPTs were lumped together as subsequent SPTs in nine studies (Hu et al. 2015; Nagasawa et al. 2000; Chen et al. 2019; Coyte et al. 2014; Komatsu et al. 2019; Levi et al. 1999; Takigawa et al. 2006; Shimizu et al. 2001; Fink-Neuboeck et al. 2020) and varying definitions were used for synchronous and metachronous in eight studies (Kumagai et al. 2001; Abdel-Rahman and Cheung 2017; Duchateau and Stokkel 2005; Haraguchi et al. 2007; Kawahara et al. 1998; Reinmuth et al. 2013; Su et al. 2017; Teppo et al. 2001); (2) the retrospective study design with limited information regarding the detection method of SPTs and lack of long-term follow-up data in most included studies; (3) both ESCC and LC often remain asymptomatic for a long time and, therefore, are frequently detected in advanced stages; (4) high heterogeneity between the included studies. These limitations in the methodology of included studies resulted in rather low prevalence rates of SPTs.

In conclusion, this meta-analysis showed that patients with ESCC and LC have an increased risk of developing SPTs in the lungs and esophagus. However, based on the rather low SPT prevalence rates found in this systematic review, screening cannot be recommended. Further research focusing on risk stratification for subgroups of patients with ESCC and LC might reveal subgroups with higher risks, potentially making screening more worthwhile.

## Supplementary Information

Below is the link to the electronic supplementary material.

Supplementary file1 (DOCX 660 KB)
