# Supplementary material for: Prevalence of lung tumors in patients with esophageal squamous cell carcinoma and vice versa: a systematic review and meta-analysis
Source: J Cancer Res Clin Oncol. 2022 Jun 23;149(5):1811–23. doi: 10.1007/s00432-022-04103-0 (PMC10097754; doi:10.1007/s00432-022-04103-0)
Supplement: Supplementary file 1 — Supplementary file1 (DOCX 660 KB) [file 432_2022_4103_MOESM1_ESM.docx]

**Supplementary Material**

**Table 1.** Quality assessment of included studies according to the Newcastle-Ottawa scale for cohort studies.

| **Author** ^ref^ | **Year** | **Selection** | **Comparability** | **Exposure/outcome** | **Total (9/9)** |
| --- | --- | --- | --- | --- | --- |
| Van de Ven et al.^8^ | 2020 | ★★★★ | ★ | ★★★ | 8 |
| Yoshida et al.^22^ | 2020 | ★★★★ | ★★ | ★ | 7 |
| Chen et al.^33^ | 2019 | ★★★★ | ★ | ★ | 6 |
| Yamaguchi et al.^25^ | 2018 | ★★★ | ★ | ★★★ | 7 |
| Otowa et al.^26^ | 2016 | ★★★★ | ★★ | ★★★ | 9 |
| Hu et al.^23^ | 2015 | ★★★★ | ★★ | ★★★ | 9 |
| Lee et al.^24^ | 2013 | ★★★★ | ★★ | ★★★ | 9 |
| Chuang et al.^34^ | 2008 | ★★★★ | ★ | ★ | 6 |
| Natsugoe et al.^27^ | 2005 | ★★★★ | ★★ | ★★★ | 9 |
| Motoyama et al. ^21^ | 2003 | ★★★★ | ★ | ★★★ | 9 |
| Kokawa et al^29^ | 2001 | ★★★★ | ★★ | ★ | 7 |
| Kumagai et al.^28^ | 2001 | ★★★★ | ★★ | ★ | 7 |
| Nagasawa et al.^30^ | 2000 | ★★★★ | ★★ | ★ | 7 |
| Ribeiro Jùnior et al.^35^ | 1999 | ★★★★ | ★ | ★ | 6 |
| Poon et al.^20^ | 1998 | ★★★★ | ★★ | ★★★ | 9 |
| Voormolen et al.^31^ | 1995 | ★★★★ | ★ | ★★★ | 8 |
| Fekete et al.^32^ | 1994 | ★★ | ★ | ★ | 4 |
| Fogel et al.^36^ | 1985 | ★★ | ★ | ★ | 4 |
| Fitzpatrick et al.^37^ | 1984 | ★★★★ | ★ | ★ | 6 |
| Fink-Neuboeck et al.^57^ | 2020 | ★★★★ | ★★ | ★★ | 8 |
| Komatsu et al.^47^ | 2019 | ★★★★ | ★★ | ★★★ | 9 |
| Faehling et al.^42^ | 2018 | ★★★★ | ★ | ★★★ | 8 |
| Abdel-Rahman et al.^38^ | 2017 | ★★★★ | ★ | ★ | 6 |
| Shan et al.^51^ | 2017 | ★★★★ | ★ | ★ | 6 |
| Su et al.^53^ | 2017 | ★★★★ | ★ | ★★ | 7 |
| Li et al.^49^ | 2015 | ★★★★ | ★ | ★ | 6 |
| Coyte et al.^40^ | 2014 | ★★★★ | ★ | ★ | 6 |
| Reinmuth et al.^50^ | 2013 | ★★★★ | ★ | ★★★ | 8 |
| Son et al.^52^ | 2013 | ★★★★ | ★★ | ★ | 7 |
| Chuang et al.^39^ | 2010 | ★★★★ | ★ | ★ | 6 |
| Haraguchi et al.^43^ | 2007 | ★★★★ | ★★ | ★ | 7 |
| Takigawa et al.^54^ | 2006 | ★★★ | 0 | ★★★ | 6 |
| Duchateau et al.^41^ | 2005 | ★★★★ | ★ | ★ | 6 |
| Shimizu et al.^56^ | 2001 | ★★★★ | 0 | ★★★ | 7 |
| Teppo et al.^55^ | 2001 | ★★★★ | ★ | ★★ | 7 |
| Kaneko et al.^45^ | 1999 | ★★★★ | ★ | ★★ | 7 |
| Levi et al.^48^ | 1999 | ★★★★ | ★ | ★★★ | 8 |
| Kawahara et al.^46^ | 1998 | ★★★★ | 0 | ★★★ | 7 |
| Hsieh et al.^44^ | 1997 | ★★★★ | ★ | ★ | 6 |

**Table 2.** Study characteristics and quality assessment of included studies performed in patients with ESCC.

| **Author** ^ref^ | **Year** | **Country** | **Study design** | **ESCC, n** | **LSPTs, n (%)** | **Time to SPTs investigated** | | | **Quality (NOS)** |
| --- | --- | --- | --- | --- | --- | --- | --- | --- | --- |
|  |  |  |  |  |  | **History of LC** | **Synchronous LSPTs** | **Metachronous LSPTs** |  |
| Van de Ven et al.^8^ | 2020 | Netherlands | Retro | 9,058 | 219 (2.4) | - | + | + | 8/9 |
| Yoshida et al.^22^ | 2020 | Japan | Retro | 766 | 22 (2.9) | + | + | + | 7/9 |
| Chen et al.^33^ | 2019 | USA | Retro | 14,540 | 229 (1.6) | - | +^b^ | | 6/9 |
| Yamaguchi et al.^25^ | 2018 | Japan | Retro | 185 | 5 (2.7) | - | - | + | 7/9 |
| Otowa et al.^26^ | 2016 | Japan | Retro | 273 | 6 (2.2) | + | + | + | 9/9 |
| Hu et al.^23^ | 2015 | China | Retro | 512 | 13 (2.5)^a^ | - | +^b^ | | 9/9 |
| Lee et al.^24^ | 2013 | Korea | Retro | 601 | 18 (3.0) | + | + | + | 9/9 |
| Chuang et al.^34^ | 2008 | World | Retro | 30,121 | 338 (1.1) | + | + | + | 6/9 |
| Natsugoe et al.^27^ | 2005 | Japan | Retro | 652 | 13 (2.0) | + | + | + | 9/9 |
| Motoyama et al.^21^ | 2003 | Japan | Retro (1972-1988)  Pro (1989-2001) | 325 | 9 (2.8) | - | - | + | 9/9 |
| Kokawa et al.^29^ | 2001 | Japan | Retro | 368 | 13 (3.5) | + | + | + | 7/9 |
| Kumagai et al.^28^ | 2001 | Japan | Retro | 733 | 5 (0.7) | + | + | +^c^ | 7/9 |
| Nagasawa et al.^30^ | 2000 | Japan | Retro | 268 | 3 (1.1) | + | +^b^ | | 7/9 |
| Ribeiro Jùnior et al.^35^ | 1999 | Brazil | Retro | 264 | 2 (0.8) | + | + | + | 6/9 |
| Poon et al.^20^ | 1998 | China | Pro | 1,055 | 8 (0.8) | + | + | + | 9/9 |
| Voormolen et al.^31^ | 1995 | Netherlands | Retro | 242 | 3 (1.2) | + | + | + | 8/9 |
| Fekete et al.^32^ | 1994 | France | Retro | 1,294 | 39 (3.0) | + | + | + | 4/9 |
| Fogel et al.^36^ | 1985 | USA | Retro | 198 | 2 (1.0) | + | + | + | 4/9 |
| Fitzpatrick et al.^37^ | 1984 | Canada | Retro | 1,469 | 6 (0.4) | + | + | + | 6/9 |
| ESCC, esophageal squamous cell carcinoma; LSPT, lung second primary tumor; NOS, Newcastle-Ottawa Scale quality assessment for cohort studies; Pro, prospective; Retro, retrospective; SPTs, second primary tumor; USA, united States of America.  ^a^ Hu et al. excluded all lung squamous cell carcinoma (n=11), which occurred within the first 5 years after the diagnosis of ESCC, as potential LSPTs.  ^b^ Synchronous and metachronous LSPTs were registered jointly as subsequent SPTs.  ^c^ Metachronous LSPTs were defined as SPTs detected more than 1 year after the diagnosis of ESCC. | | | | | | | | | |

**Table 3.** Study characteristics and quality assessment of included studies performed in patients with LC.

| **Author** ^ref^ | **Year** | **Country** | **Study design** | **LC type** | **LC, n** | **ESPTs, n (%)** | **Time to SPTs investigated** | | | **Quality (NOS)** |
| --- | --- | --- | --- | --- | --- | --- | --- | --- | --- | --- |
|  |  |  |  |  |  |  | **History of EC** | **Synchronous ESPTs** | **Metachronous ESPTs** |  |
| Fink-Neuboeck et al.^57^ | 2020 | Austria | Pro | NSCLC | 324 | 1 (0.3) | + | +^b^ | | 8/9 |
| Komatsu et al.^47^ | 2019 | Japan | Retro | NSCLC | 521 | 27 (5.2) | - | +^b^ | | 9/9 |
| Faehling et al.^42^ | 2018 | Germany | Retro | NSCLC | 1,252 | 3 (0.2) | +^a^ | + | - | 8/9 |
| Abdel-Rahman et al.^38^ | 2017 | USA | Retro | LC | 223,274 | 251 (0.1) | - | + | +^c^ | 6/9 |
| Shan et al.^51^ | 2017 | China | Retro | LC | 27,642 | 10 (0.04) | + | + | - | 6/9 |
| Su et al.^53^ | 2017 | Taiwan | Retro | LC | 18,372 | 16 (0.09) | - | - | +^c^ | 7/9 |
| Li et al.^49^ | 2015 | China | Retro | LC | 5,405 | 17 (0.3) | + | + | + | 6/9 |
| Coyte et al.^40^ | 2014 | Scotland | Retro | LC | 10,764 | 4 (0.04) | - | +^b^ | | 6/9 |
| Reinmuth et al.^50^ | 2013 | Germany | Retro | LC | 2,816 | 1 (0.04) | - | + | +^d^ | 8/9 |
| Son et al.^52^ | 2013 | Korea | Retro | NSCLC | 632 | 4 (0.6) | + | + | + | 7/9 |
| Chuang et al.^39^ | 2010 | World | Retro | LC | 258,559 | 159 (0.06) | - | + | + | 6/9 |
| Haraguchi et al.^43^ | 2007 | Japan | Retro | LC | 1,013 | 4 (0.4) | - | + | +^c^ | 7/9 |
| Takigawa et al.^54^ | 2006 | Japan | Retro | NSCLC | 90 | 2 (2.2) | - | +^b^ | | 6/9 |
| Duchateau et al.^41^ | 2005 | Netherlands | Retro | NSCLC | 860 | 2 (0.2) | + | + | +^d^ | 6/9 |
| Shimizu et al.^56^ | 2001 | Japan | Pro | LC | 32 | 1 (3.1) | - | +^b^ | | 7/9 |
| Teppo et al.^55^ | 2001 | Finland | Retro | LC | 77,548 | 28 (0.04) | - | + | +^e^ | 7/9 |
| Kaneko et al.^45^ | 1999 | Japan | Retro | LC | 6,935 | 28 (0.4) | - | + | - | 7/9 |
| Levi et al.^48^ | 1999 | Switzerland | Retro | LC | 5,794 | 5 (0.09) | - | +^b^ | | 8/9 |
| Kawahara et al.^46^ | 1998 | Japan | Retro | SCLC | 70 | 2 (2.9) | - | - | +^d^ | 7/9 |
| Hsieh et al.^44^ | 1997 | Taiwan | Retro | LC | 6,412 | 10 (0.2) | + | + | + | 6/9 |
| ESPT, esophageal second primary tumor; LC, lung cancer; NOS, Newcastle-Ottawa Scale quality assessment for cohort studies; NSCLC, non-small cell lung cancer; SCLC, small cell lung cancer; Pro, prospective; Retro, retrospective; SPT, second primary tumor; USA, United States of America.  ^a^ History of EC was defined as the detection of EC more than 3 months before the diagnosis of LC.  ^b^ Synchronous and metachronous ESPTs were registered jointly as subsequent SPTs.  ^c^ Metachronous ESPTs were defined as SPTs detected more than 1 year after the diagnosis of LC.  ^d^ Metachronous ESPTs were defined as SPTs detected more than 2 years after the diagnosis of LC.  ^e^ Metachronous ESPTs were defined as SPTs detected during follow-up (i.e. after first admission for LC). | | | | | | | | | | |

**Figure 1.** Funnel plots to assess the risk of publication bias.


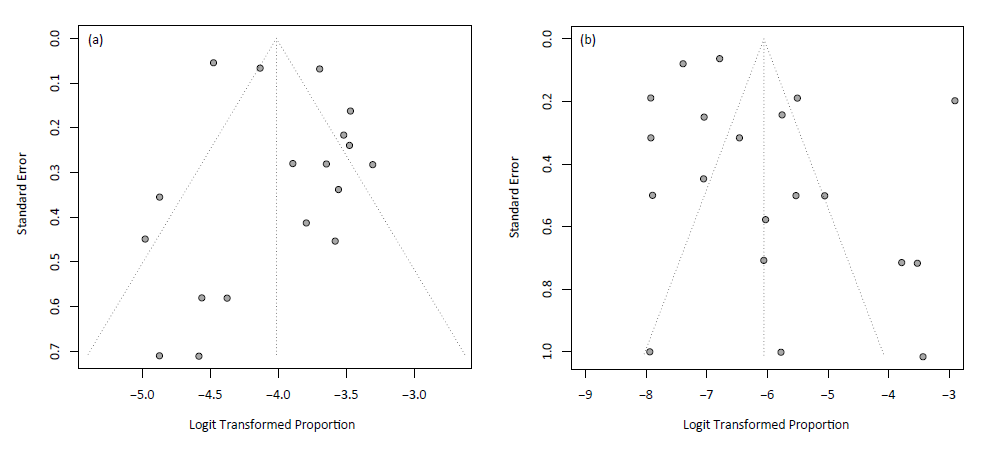
The risk for publication bias for studies (a) performed in patients with ESCC to detect LSPTs (*P* = 0.11) and (b) performed in patients with LC to detect ESPTs (*P* = 0.16).

ESCC, esophageal squamous cell carcinoma; ESPTs, esophageal second primary tumors; LC, lung cancer; LSPTs, lung second primary tumors.

**Figure 2**. Overview of the prevalence of LSPTs in patients with ESCC in Asian and non-Asian countries.


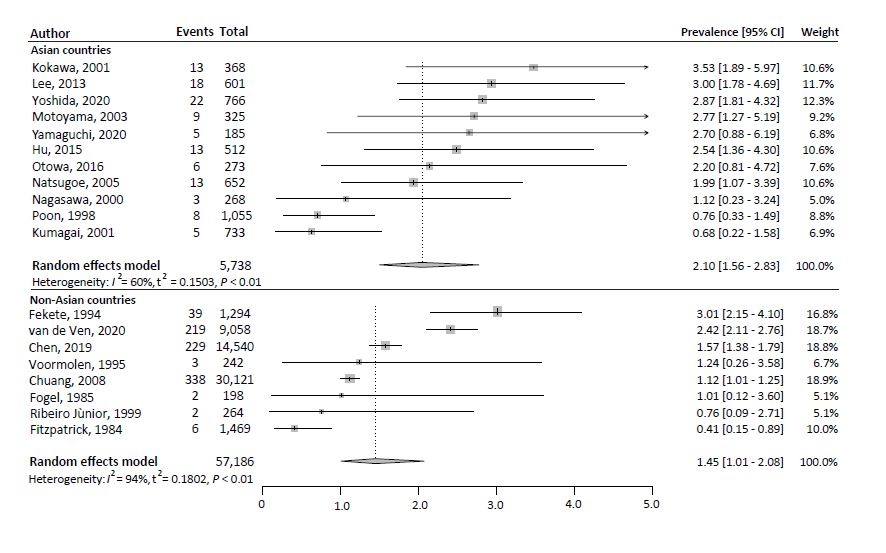


CI, confidence interval; ESCC, oesophageal squamous cell carcinoma; LSPT, lung second primary tumour; *I*^2^, inconsistency index; τ^2^, tau-squared represents the extent of variation among the effects observed in different studies.

^a^ Hu et al. excluded all lung squamous cell carcinoma (n=11), which occurred within the first 5 years after the diagnosis of ESCC, as potential LSPTs.^23^

**Figure 3**. Overview of the prevalence of LSPTs in patients with ESCC during recent decades.


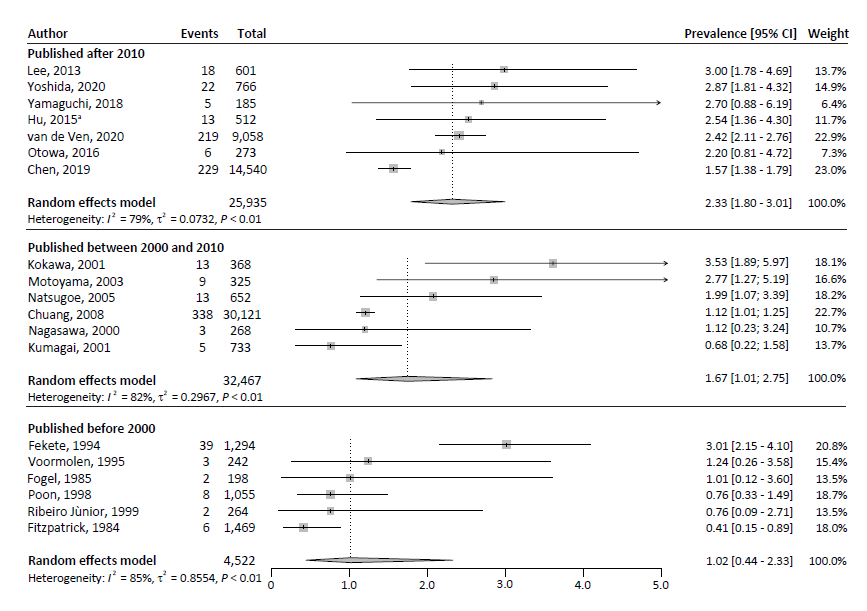


CI, confidence interval; ESCC, oesophageal squamous cell carcinoma; LSPT, lung second primary tumour; *I*^2^, inconsistency index; τ^2^, tau-squared represents the extent of variation among the effects observed in different studies.

^a^ Hu et al. excluded all lung squamous cell carcinoma (n=11), which occurred within the first 5 years after the diagnosis of ESCC, as potential LSPTs.^23^

**Figure 4**. Overview of the prevalence of ESPTs in patients with LC in Asian and non-Asian countries.


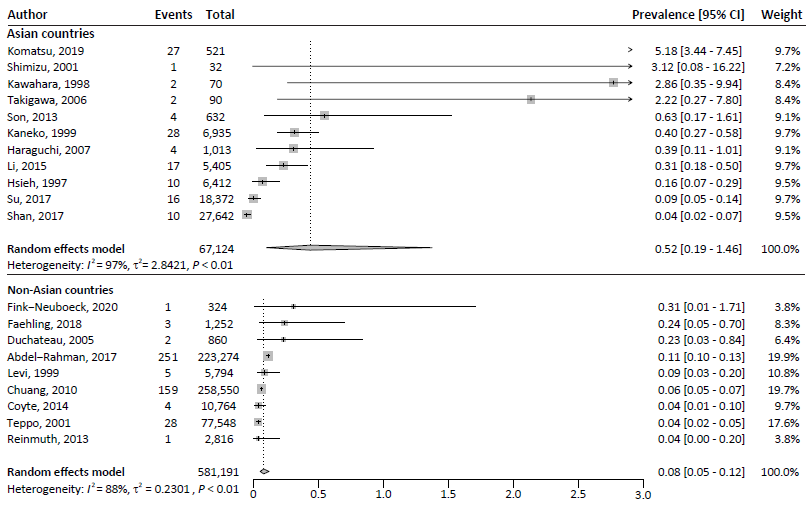


CI, confidence interval; ESPT, oesophageal second primary tumour; LC, lung cancer; *I*^2^, inconsistency index; τ^2^, tau-squared represents the extent of variation among the effects observed in different studies.

**Figure 5**. Overview of the prevalence of ESPTs in patients with LC during recent decades.


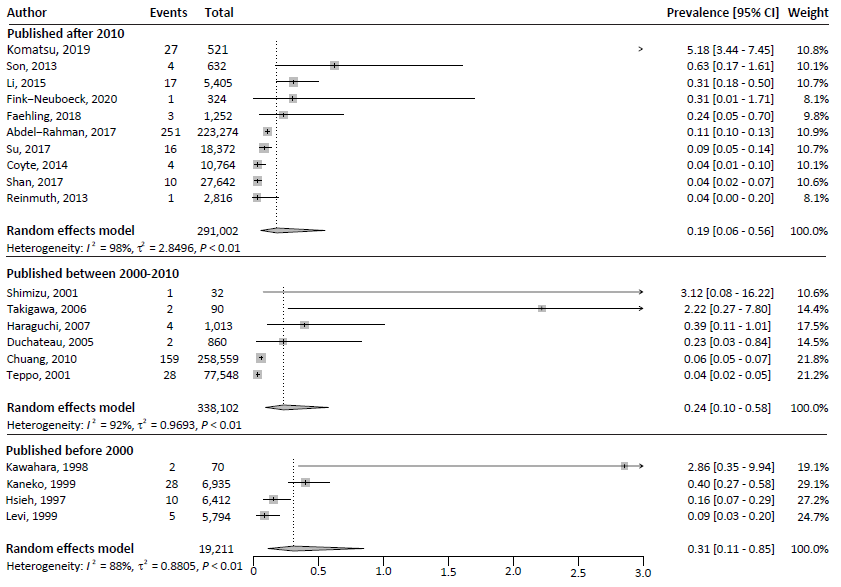


CI, confidence interval; ESPT, oesophageal second primary tumour; LC, lung cancer; *I*^2^, inconsistency index; τ^2^, tau-squared represents the extent of variation among the effects observed in different studies.


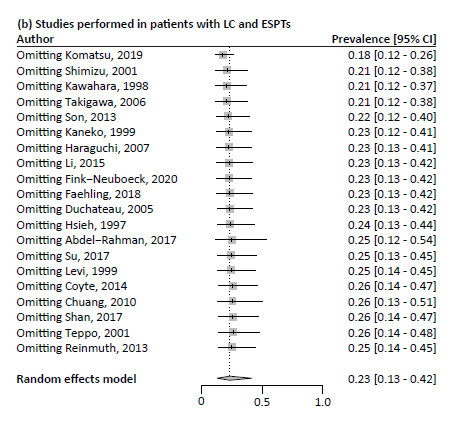

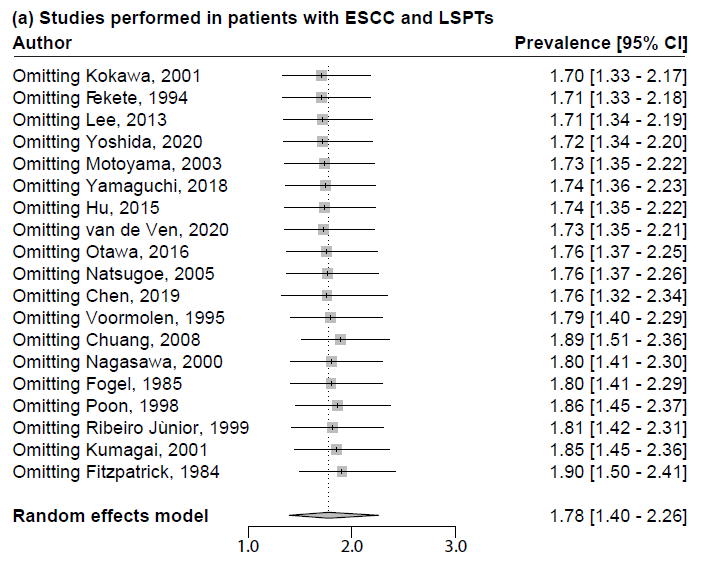
**Figure 6**. Excessive influence analysis of the included studies.

ESCC, esophageal squamous cell carcinoma; ESPT, esophageal second primary tumor; LC, lung cancer; LSPT, lung second primary tumor.

**Appendix 1.** The full search strategy

**Embase**

('second cancer'/de OR (((Metachronous OR Synchronous) NEAR/6 (tumo* OR malignan* OR carcin* OR neoplas* OR cancer*)) OR ((Second OR Multiple OR double OR triple OR quadruple OR quintuple OR subsequen* OR Simultan*) NEAR/3 (primar*) NEAR/6 (tumo* OR malignan* OR carcin* OR neoplas* OR cancer*))):ab,ti,kw) AND ('esophagus tumor'/exp OR 'lung tumor'/exp OR ((esophag* OR oesophag* OR lung OR pulmonar* OR upper-aerodigest* OR upper-digest*) NEAR/6 (tumo* OR cancer* OR neoplas*)):ab,ti,kw) NOT [conference abstract]/lim NOT ([animals]/lim NOT [humans]/lim) AND [English]/lim

**Medline**

(Neoplasms, Second Primary/ OR Neoplasms, Multiple Primary/ OR (((Metachronous OR Synchronous) ADJ6 (tumo* OR malignan* OR carcin* OR neoplas* OR cancer*)) OR ((Second OR Multiple OR double OR triple OR quadruple OR quintuple OR subsequen* OR Simultan*) ADJ3 (primar*) ADJ6 (tumo* OR malignan* OR carcin* OR neoplas* OR cancer*))).ab,ti,kf.) AND (exp Esophageal Neoplasms/ OR exp Lung Neoplasms/ OR ((esophag* OR oesophag* OR lung OR pulmonar* OR upper-aerodigest* OR upper-digest*) ADJ6 (tumo* OR cancer* OR neoplas*)).ab,ti,kf.) NOT (exp animals/ NOT humans/) AND english.la.

**Web of science**

TS=(((((Metachronous OR Synchronous) NEAR/5 (tumo* OR malignan* OR carcin* OR neoplas* OR cancer*)) OR ((Second OR Multiple OR double OR triple OR quadruple OR quintuple OR subsequen* OR Simultan*) NEAR/2 (primar*) NEAR/5 (tumo* OR malignan* OR carcin* OR neoplas* OR cancer*)))) AND (((esophag* OR oesophag* OR lung OR pulmonar* OR upper-aerodigest* OR upper-digest*) NEAR/5 (tumo* OR cancer* OR neoplas*)))) AND DT=(Article OR Review OR Letter OR Early Access) AND LA=(english)

**Cochrane**

((((Metachronous OR Synchronous) NEAR/6 (tumo* OR malignan* OR carcin* OR neoplas* OR cancer*)) OR ((Second OR Multiple OR double OR triple OR quadruple OR quintuple OR subsequen* OR Simultan*) NEAR/3 (primar*) NEAR/6 (tumo* OR malignan* OR carcin* OR neoplas* OR cancer*))):ab,ti,kw) AND (((esophag* OR oesophag* OR lung OR pulmonar* OR upper-aerodigest* OR upper-digest*) NEAR/6 (tumo* OR cancer* OR neoplas*)):ab,ti,kw)

**Google scholar**

"Metachronous|Synchronous tumors|malignancies|neoplasms|cancers"|"Second|Multiple|double|triple primary tumor|malignancy|carcinoma|neoplasm|cancer" esophagus|oesophagus|esophageal|oesophageal|lung|pulmonary incidence|prevalence

'Metachronous|Synchronous tumors|malignancies|neoplasms|cancers'|'Second|Multiple|double|triple primary tumor|malignancy|carcinoma|neoplasm|cancer' esophagus|oesophagus|esophageal|oesophageal|lung|pulmonary incidence|prevalence
